# Supplementary material for: Molecular engineering towards efficientwhite-light-emitting perovskite
Source: Nat Commun. 2021 Aug 12;12:4890. doi: 10.1038/s41467-021-25132-2 (PMC8361204; doi:10.1038/s41467-021-25132-2)
Supplement: Supplementary file 1 — Supplementary Information [file 41467_2021_25132_MOESM1_ESM.pdf]

Supplementary Information

Molecular Engineering towards Efficient White-Light-Emitting Perovskite

Mingming Zhang[1,7], Lili Zhao[2,7], Jiahao Xie[3,7], Qian Zhang[1,7], Xiaoyu Wang[3], Najma Yaqoob[4], Zhengmao Yin[5], Payam Kaghazchi[4], San Zhang[6], Hua Li[2], Chunfeng Zhang[6], Lei Wang[1], Lijun Zhang[3]*, Weigao Xu[2]* & Jun Xing[1]*

[1]Key Laboratory of Eco-Chemical Engineering, Ministry of Education, College of Chemistry and Molecular Engineering, Qingdao University of Science & Technology, Qingdao 266042, China.

[2]Key Laboratory of Mesoscopic Chemistry, Ministry of Education, School of Chemistry and Chemical Engineering, Nanjing University, Nanjing 210023, China.

[3]State Key Laboratory of Integrated Optoelectronics, Key Laboratory of Automobile Materials of MOE, School of Materials Science and Engineering, Jilin University, Changchun 130012, China.

[4]Forschungszentrum Jülich GmbH, Institute of Energy and Climate Research, Materials Synthesis and Processing (IEK-1), Wilhelm-Johnen-Straße, 52425 Jülich, Germany.

[5]School of Materials Science and Technology, Qingdao University of Science & Technology, Qingdao 266042, China.

[6]National Laboratory of Solid State Microstructures, School of Physics and Collaborative Innovation Center of Advanced Microstructures, Nanjing University, Nanjing, China

[7]These authors contributed equally: Mingming Zhang, Lili Zhao, Jiahao Xie, Qian Zhang.

*correspondence: lijun_zhang@jlu.edu.cn, xuwg@nju.edu.cn and xingjun@qust.edu.cn

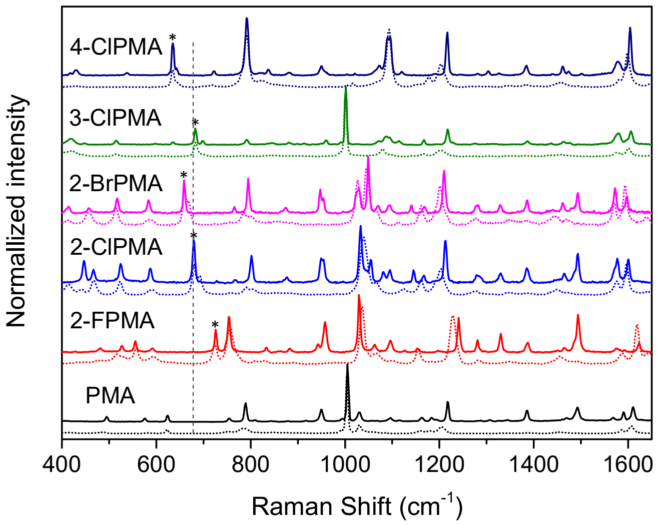

Supplementary Fig. 1. Raman spectra of perovskite $(PMA)_2PbBr_4$, $(2\text{-}FPMA)_2PbBr_4$, $(2\text{-}ClPMA)_2PbBr_4$, $(2\text{-}BrPMA)_2PbBr_4$, $(3\text{-}ClPMA)_2PbBr_4$, $(4\text{-}ClPMA)_2PbBr_4$ (solid lines) and their corresponding alkylamines precursors purchased from Aladdin (dashed lines).

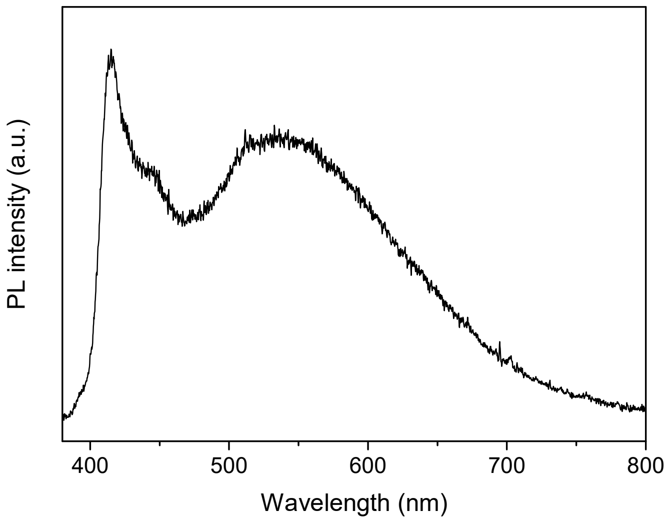

Supplementary Fig. 2. PL spectrum of $(2\text{-ClPMA})_2PbBr_4$ synthesized by fast reprecipitation method.

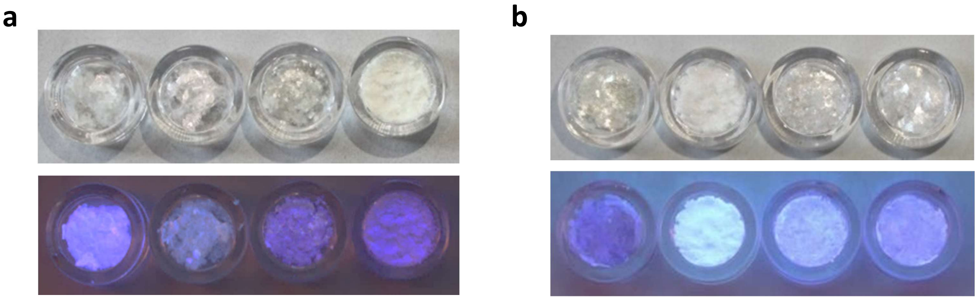

Supplementary Fig. 3. Digital images of (a) $(PMA)_2PbBr_4$, $(2\text{-}FPMA)_2PbBr_4$, $(3\text{-}FPMA)_2PbBr_4$, $(4\text{-}FPMA)_2PbBr_4$ and (b) $(PMA)_2PbBr_4$, $(2\text{-}BrPMA)_2PbBr_4$, $(3\text{-}BrPMA)_2PbBr_4$, $(4\text{-}BrPMA)_2PbBr_4$ crystals under ambient light (upper) and UV light (lower).

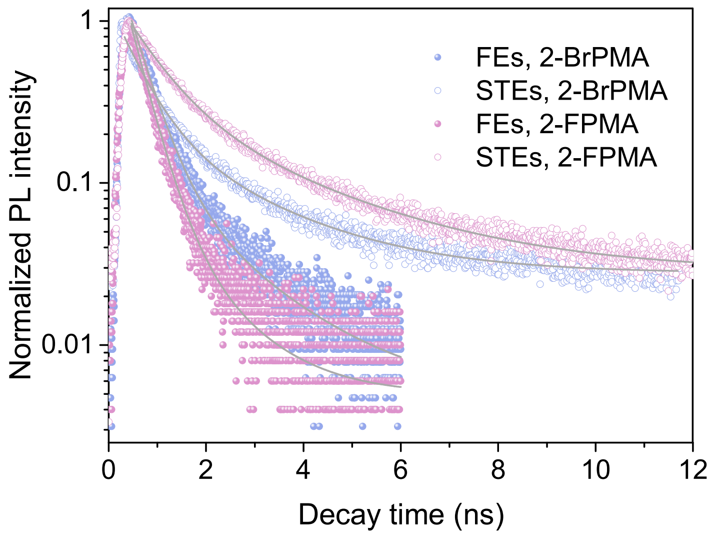

Supplementary Fig. 4. PL decay profiles from FEs and STEs of $(2\text{-FPMA})_2PbBr_4$ and $(2\text{-BrPMA})_2PbBr_4$.

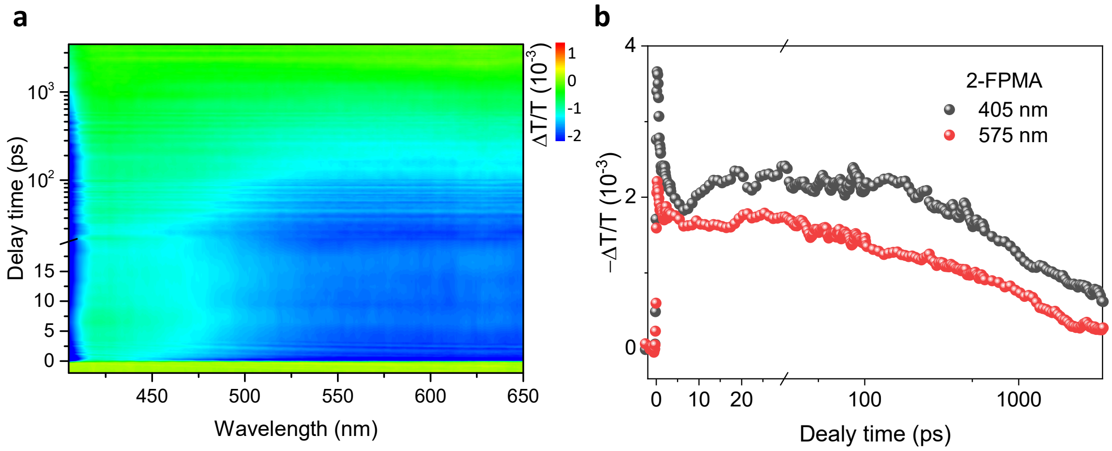

Supplementary Fig. 5. (a) 2D color plots of TA spectra of $(2\text{-FPMA})_2PbBr_4$. (b) TA time delay probed at wavelengths of 405 and 575 nm, respectively.

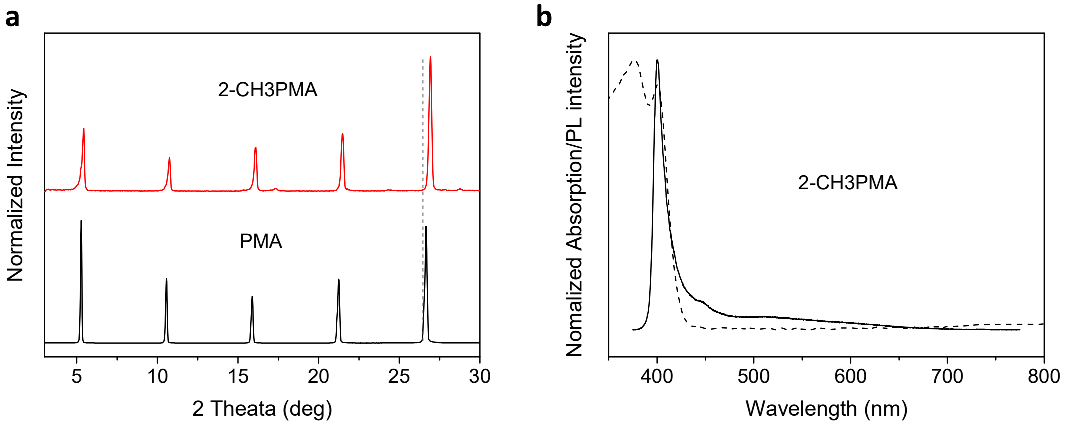

Supplementary Fig. 6. (a) Powder XRD patterns of perovskites $(PMA)_2PbBr_4$ and $(2\text{-}CH_3PMA)_2PbBr_4$. (b) Absorption (dashed line) and PL spectrum (solid line) of $(2\text{-}CH_3PMA)_2PbBr_4$.

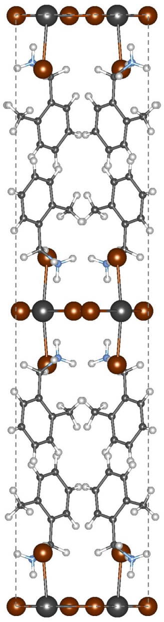

Supplementary Fig. 7. The atomic structure of perovskite $(2\text{-}CH_3PMA)_2PbBr_4$.

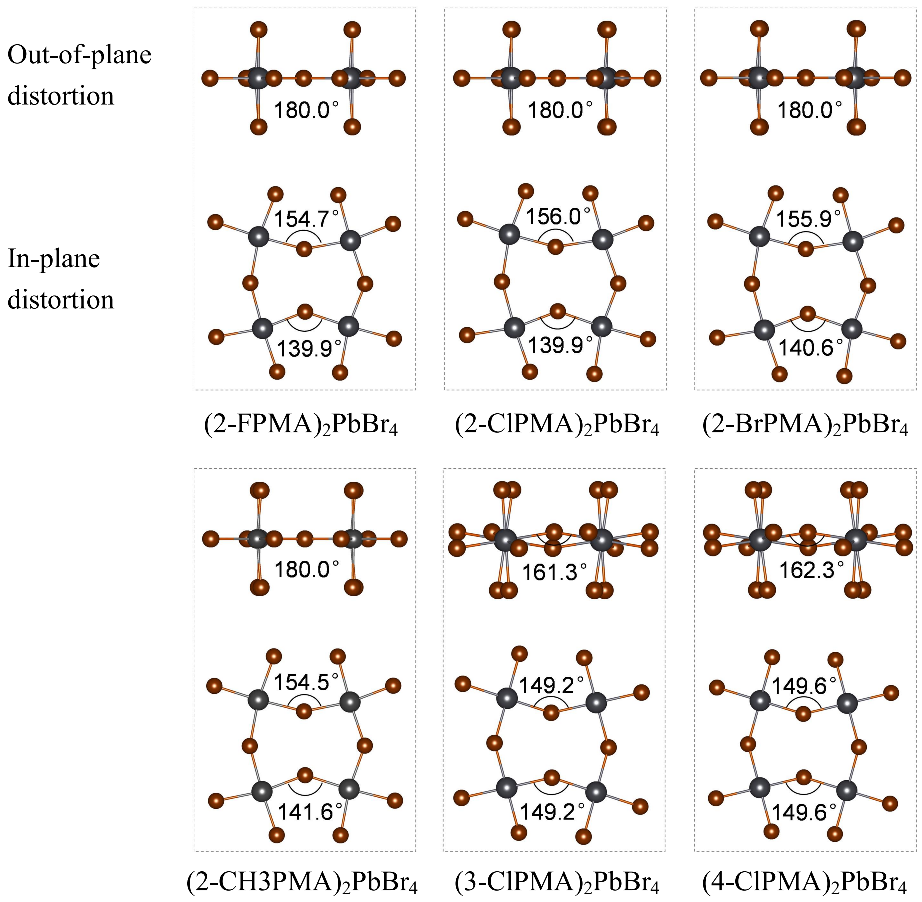

Supplementary Fig. 8. The structure of out-of-plane and in-plane distortion of perovskites.

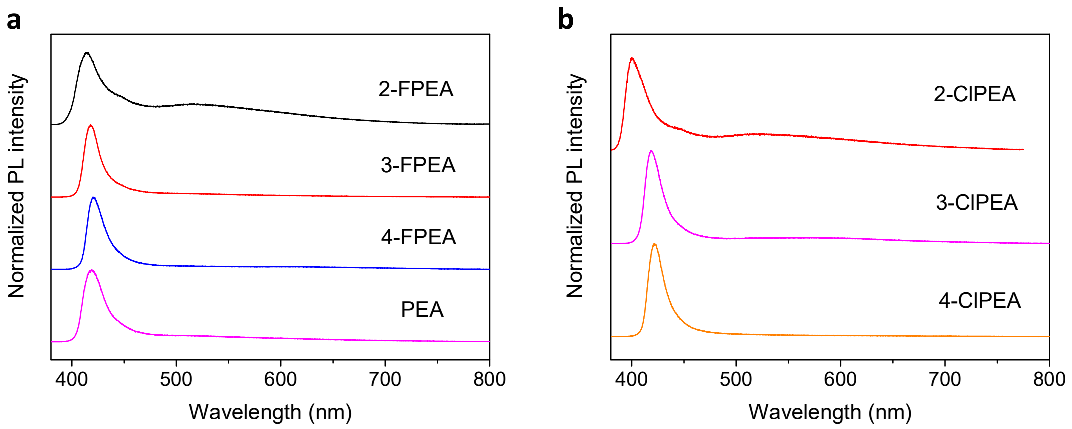

Supplementary Fig. 9. PL spectra of perovskites (a) $(PEA)_2PbBr_4$, $(2\text{-}FPEA)_2PbBr_4$, $(3\text{-}FPEA)_2PbBr_4$, $(4\text{-}FPEA)_2PbBr_4$ and (b) $(2\text{-}ClPEA)_2PbBr_4$, $(3\text{-}ClPEA)_2PbBr_4$, $(4\text{-}ClPEA)_2PbBr_4$.

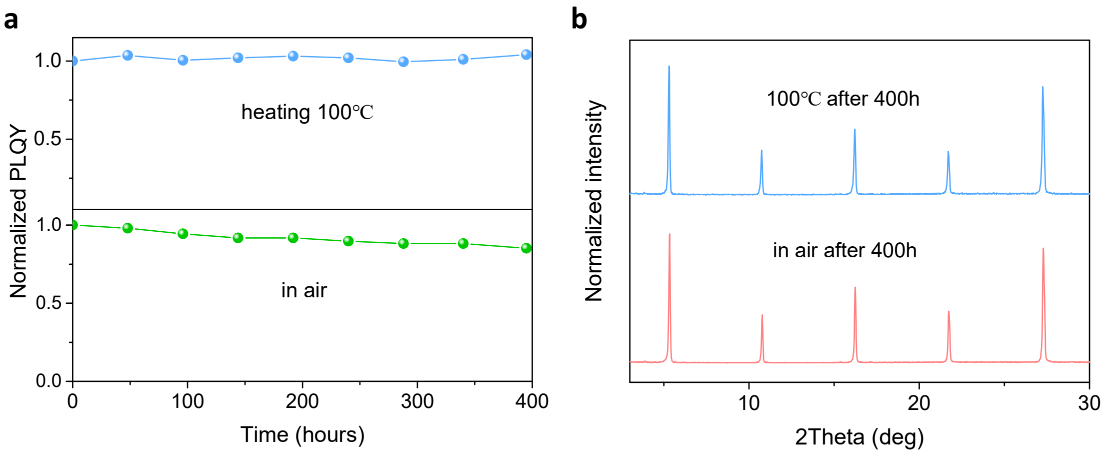

Supplementary Fig. 10. (a) Stability of the perovskite $(2\text{-ClPMA})_2PbBr_4$ against continuous heating at 100 °C on hotplate in N_2 glovebox and exposing in air with average temperature of 25 °C and humidity of 40%. (b) XRD patterns of the perovskite $(2\text{-ClPMA})_2PbBr_4$ after stability measurements.

Supplementary Table 1. Crystal lattice constants of perovskites $(PMA)_2PbBr_4$. Data is from reference Inorg. Chem. 56, 9291-9302 (2017).

$(PMA)_2PbBr_4$ (orthorhombic)	**a**	**b**	**c**
	33.35 Å	8.15 Å	8.12 Å
	**α**	**β**	**γ**
	90°	90°	90°

Supplementary Table 2. Crystal data and structure refinement for $(2\text{-FPMA})_2PbBr_4$, $(2\text{-ClPMA})_2PbBr_4$ and $(3\text{-ClPMA})_2PbBr_4$.

**Compound**	**2-FPMA**	**2-ClPMA**	**3-ClPMA**
Empirical formula	$C_{28}H_{36}Br_8F_4N_4Pb_2$	$C_7H_9Br_2ClNPb_{0.5}$	$C_7H_9Br_2ClNPb_{0.5}$
Formula weight	1558.27	406.02	406.02
Temperature/K	293	293	293
Crystal system	orthorhombic	orthorhombic	monoclinic
Space group	$Cmc2_1$	Pnma	$P2_1/c$
a/Å	33.3960(4)	8.02372(16)	17.6670(5)
b/Å	8.19720(10)	32.5193(6)	7.83158(18)
c/Å	8.03670(10)	8.39334(15)	8.19448(17)
α/°	90	90	90
β/°	90	90	97.806(2)
γ/°	90	90	90
Volume/Å^3	2200.08(5)	2190.04(7)	1123.28(5)
Z	2	8	4
ρ_{calc}g/cm^3	2.352	2.463	2.401
μ/mm^{-1}	23.565	25.771	25.122
F(000)	1424	1488	744
Radiation	CuKα (λ = 1.54184)	CuKα (λ = 1.54184)	CuKα (λ = 1.54184)
2θ range for data collection/°	10.596 to 141.362	10.882 to 134.076	10.108 to 141.368
Index ranges	$-40 \le h \le 40$, $-7 \le k \le 9$, $-8 \le l \le 9$	$-8 \le h \le 9$, $-38 \le k \le 38$, $-9 \le l \le 10$	$-21 \le h \le 21$, $-5 \le k \le 9$, $-9 \le l \le 9$
Reflections collected	7295	4173	3936
Independent reflections	2034 [R_{int} = 0.0337, R_{sigma} = 0.0244]	1962 [R_{int} = 0.0412, R_{sigma} = 0.0396]	2043 [R_{int} = 0.0421, R_{sigma} = 0.0446]
Data/restraints/parameters	2034/1/98	1962/0/110	2043/0/107
Goodness-of-fit on F^2	1.055	1.039	1.155
Final R indexes [I>=2σ (I)]	R_1 = 0.0380, wR_2 = 0.0973	R_1 = 0.0564, wR_2 = 0.1456	R_1 = 0.0808, wR_2 = 0.2225
Final R indexes [all data]	R_1 = 0.0383, wR_2 = 0.0978	R_1 = 0.0588, wR_2 = 0.1494	R_1 = 0.0871, wR_2 = 0.2344
Largest diff. peak/hole / e Å^{-3}	2.25/-1.60	5.74/-1.72	8.38/-3.16

Supplementary Table 3. Crystal data and structure refinement for $(4\text{-ClPMA})_2PbBr_4$, $(2\text{-BrPMA})_2PbBr_4$ and $(2\text{-CH}_3\text{PMA})_2PbBr_4$.

**Compound**	**4-ClPMA**	**2-BrPMA**	**2-CH$_3$PMA**
Empirical formula	$C_7H_9Br_2ClNPb_{0.5}$	$C_7H_9Br_3NPb_{0.5}$	$C_8H_{12}Br_2NPb_{0.5}$
Formula weight	406.02	450.48	385.60
Temperature/K	293	293	112
Crystal system	orthorhombic	orthorhombic	orthorhombic
Space group	Pccn	Pnma	Pnma
a/Å	7.91038(18)	8.07030(18)	8.09206(12)
b/Å	35.0125(7)	32.5348(9)	32.7509(4)
c/Å	8.13179(18)	8.41533(16)	8.21055(12)
α/°	90	90	90
β/°	90	90	90
γ/°	90	90	90
Volume/Å^3	2252.20(8)	2209.58(9)	2175.98(5)
Z	8	8	8
ρ_{calc}g/cm^3	2.395	2.708	2.354
μ/mm^{-1}	25.059	27.556	15.099
F(000)	1488	1632	1424
Radiation	CuKα (λ = 1.54184)	CuKα (λ = 1.54184)	CuKα (λ = 1.54184)
2θ range for data collection/°	11.468 to 134.126	10.86 to 134.14	11.11 to 141.156
Index ranges	$-7 \le h \le 9$<br>$-40 \le k \le 41$<br>$-4 \le l \le 9$	$-9 \le h \le 8$<br>$-18 \le k \le 38$<br>$-10 \le l \le 7$	$-9 \le h \le 9$<br>$-39 \le k \le 39$<br>$-5 \le l \le 10$
Reflections collected	4379	4313	10468
Independent reflections	1972 [R_{int} = 0.0753, R_{sigma} = 0.0619]	1972 [R_{int} = 0.0515, R_{sigma} = 0.0431]	2073 [Rint = 0.0405, Rsigma = 0.0226]
Data/restraints/parameters	1972/0/107	1972/0/110	2073/0/111
Goodness-of-fit on F^2	1.055	1.249	1.181
Final R indexes [I>=2σ (I)]	R_1 = 0.0885, wR_2 = 0.2313	R_1 = 0.0994, wR_2 = 0.2755	R_1 = 0.0411, wR_2 = 0.1144
Final R indexes [all data]	R_1 = 0.0953, wR_2 = 0.2481	R_1 = 0.1026, wR_2 = 0.2835	R_1 = 0.0413, wR_2 = 0.1147
Largest diff. peak/hole / e Å^{-3}	3.37/-4.46	9.07/-3.17	4.53/-1.47

Supplementary Table 4. Interlayer distances of the layered perovskites calculated from powder XRD.

Sample	interlayer distance (Å)
$(PMA)_2PbBr_4$	16.71
$(2\text{-}FPMA)_2PbBr_4$	16.72
$(3\text{-}FPMA)_2PbBr_4$	16.70
$(4\text{-}FPMA)_2PbBr_4$	17.13
$(2\text{-}ClPMA)_2PbBr_4$	16.27
$(3\text{-}ClPMA)_2PbBr_4$	17.51
$(4\text{-}ClPMA)_2PbBr_4$	17.56
$(2\text{-}BrPMA)_2PbBr_4$	16.25
$(3\text{-}BrPMA)_2PbBr_4$	17.93
$(4\text{-}BrPMA)_2PbBr_4$	17.71

Supplementary Table 5. The performance of reported low-dimensional hybrid perovskites with BWL emission.

Compositions	PLQY (%)	CRI	CIE	Ref
(N-MEDA)$PbBr_4$	0.5	82	(0.36, 0.41)	J. Am. Chem. Soc. 2014, 136, 1718
(EDBE)$PbBr_4$	9	84	(0.39, 0.42)	J. Am. Chem. Soc. 2014, 136, 13154
(EDBE)$PbCl_4$	2	81	(0.33, 0.39)	
$(PEA)_2PbCl_4$	< 1	84	(0.37, 0.42)	Chem. Mater. 2017, 29, 3947
$(EA)_4Pb_3Cl_{10}$	/	66	(0.27, 0.39)	J. Am. Chem. Soc. 2017, 139, 11956
$(EA)_4Pb_3Cl_{9.5}Br_{0.5}$	/	83	(0.30, 0.35)	
(CyBMA)$PbBr_4$	1.5	/	(0.23, 0.29)	ChemSusChem 2017, 10, 3765
$C_4N_2H_{14}PbBr_4$	20	63	(0.21, 0.28)	Nat. Commun. 2017, 8, 14051
$(NAI)_2PbCl_4$	< 1	90	(0.36, 0.37)	J. Mater. Chem. C 2018, 6, 1171
(2meptH2)$PbBr_4$	3.37	91	(0.24, 0.23)	Chem. Commun. 2018, 54, 4053
$C_5H_{14}N_2PbCl_4\cdot H_2O$	1	94	(0.39, 0.37)	J. Mater. Chem. C 2018, 6, 6033
(BAPP)Pb_2Br_8	1.5	87	(0.37, 0.40)	Adv. Mater. 2019, 31, 1807383
(TDMP)$PbBr_4$	46	75	(0.29, 0.37)	
(TDMP)$PbCl_4$	7.5	74	/	J. Am. Chem. Soc. 2019, 141, 12619
$(C_4H_9NH_3)_2PbCl_4$	1	86	(0.37, 0.40)	Adv. Funct. Mater. 2019, 29, 1805038
(AMP)$PbCl_4$	< 1	90	(0.28, 0.34)	
(1HIEA)$PbBr_4$	/	73	(0.32, 0.41)	J. Mater. Chem. C 2020, 8, 889
**(2-ClPMA)$_2$PbBr$_4$**	**32**	**86**	**(0.30, 0.33)**	**This work**

Supplementary Table 6. The PL decay parameters of $(2\text{-FPMA})_2PbBr_4$, $(2\text{-ClPMA})_2PbBr_4$, $(3\text{-ClPMA})_2PbBr_4$ and $(2\text{-BrPMA})_2PbBr_4$.

	$\tau_{1,STEs}$ (ns)	$\tau_{2,STEs}$ (ns)	$\tau_{1,FEs}$ (ns)	$\tau_{2,FEs}$ (ns)
$(2\text{-FPMA})_2PbBr_4$	2.72	0.73	1.30	0.36
$(2\text{-ClPMA})_2PbBr_4$	3.31	0.93	1.58	0.38
$(3\text{-ClPMA})_2PbBr_4$	3.23	0.84	1.50	0.35
$(2\text{-BrPMA})_2PbBr_4$	2.08	0.54	1.60	0.41

According to previous report (Chem. Sci. 2017, 8, 4497), the emission ratio I_{STEs}/I_{FEs} at a given temperature is related to $\Delta G_{self\text{-}trap}$ (self-trapping depth = $E_{STEs} - E_{FEs}$) at emission peak and the radiative emission rates from the STE and FEs states ($k_{r,STEs}$ and $k_{r,FEs}$).

$$\ln\left(\frac{I_{STEs}}{I_{FEs}}\right) \propto \ln\left(\frac{k_{r,STEs}}{k_{r,FEs}}\right) - \frac{\Delta G_{self\text{-}trap}}{k_B T} \quad (1)$$

Equals to

$$\ln\left(\frac{I_{STEs}}{I_{FEs}}\right) \propto \ln\left(\frac{\tau_{r,FEs}}{\tau_{r,STEs}}\right) - \frac{\Delta G_{self\text{-}trap}}{25.7\ \text{meV}}\ (T = 298\ \text{K}) \quad (2)$$

In this case, the average STEs emission energy is several hundreds of meV lower than that of FEs, implying the STEs state should be much lower than that of FEs state. So,

$$\frac{\Delta G_{self\text{-}trap}}{25.7\ \text{meV}} \gg \ln\left(\frac{1.3}{2.72}\right)((2\text{-FPMA})_2PbBr_4),\ \ln\left(\frac{1.58}{3.31}\right)((2\text{-ClPMA})_2PbBr_4),$$
$$\ln\left(\frac{1.6}{2.08}\right)((2\text{-BrPMA})_2PbBr_4)\ \text{or}\ \ln\left(\frac{1.5}{3.23}\right)((3\text{-ClPMA})_2PbBr_4)$$

Thus, I_{STEs}/I_{FEs} is dominated by the $\Delta G_{self\text{-}trap}$.

Supplementary Table 7. The average charges of $-CH_2NH_3^+$ and $PbBr_4^{2-}$ of perovskites, and their corresponding Coulomb forces.

	**PMA**	**2-FPMA**	**2-ClPMA**	**2-BrPMA**	**3-ClPMA**	**4-ClPMA**	**2-CH$_3$PMA**
**Average charges of $-CH_2NH_3^+$ / e**	0.6730	0.7652	0.7342	0.7321	0.6933	0.7080	0.6685
**Average charges of** $PbBr_4^{2-}$ / e	-1.4596	-1.4450	-1.4733	-1.4864	-1.4857	-1.4778	-1.4695
**Average distance between $-CH_2NH_3^+$ and** $PbBr_4^{2-}$ / Å	4.9854	4.9840	5.0465	5.0726	5.1347	5.1429	5.0568
**Coulomb force / 10^{-10} N**	9.1179	10.2688	9.7992	9.7561	9.0135	9.1256	8.8630

Supplementary Note 1.

Emission from permanent defects typically shows a sublinear dependence on excitation power density with a saturation of limited defect sites under high excitation intensity. For linear power dependent emission from permanent defects: excitation rate << relaxation rate.[1,2]

σ = absorption cross section, A = absorbance, ε_M = molar extinction coefficient, L = film thickness, C = concentration, N_A = Avogadro's number, q_p = laser photon flux, I = laser intensity, h = Planck's constant, c = velocity of light, λ = laser wavelength (390 nm). To obtain the absorption parameters of sample, we fabricated a $(2\text{-ClPMA})_2PbBr_4$ perovskite film by spincoating method. Its thickness is about 100 nm and absorbance at 390 nm is about 0.8.

Excitation rate = $\boldsymbol{\sigma q_p}$

$A = \varepsilon_M \times C \times L$ ($C = 0.0030$ mol/cm^3 obtained from formula units per unit cell volume)

$\varepsilon_M = A / (C \times L) = 2.67 \times 10^7$ cm^2/mol

$\sigma = \ln(10) \times (\varepsilon_M / N_A) = 1.02 \times 10^{-16}$ cm^2/ Pb^{2+}

$I = 23.5$ W/cm^2 (2.45×10^6 W/cm^2 for one pulse)

$q_p = I\lambda/hc = 4.81 \times 10^{24}$ s^{-1} cm^{-2}

$\sigma q_p = (1.02 \times 10^{-16}$ cm^2/$Pb^{2+}) \times (4.81 \times 10^{24}$ s^{-1} cm$^{-2}) \approx 5 \times 10^8$ (s^{-1}/Pb^{2+})

Relaxation rate = $\boldsymbol{N}$/(PL lifetime)

N = number of emissive defects per Pb^{2+}. PL lifetime at 298 K ~ 10^{-9} s

If emission arises from permanent material defects, PL unsaturation at laser power density of 23.5 W/cm^2 occurs only when Excitation rate << relaxation rate.

5×10^8 (s^{-1}/Pb^{2+}) $\ll N/(10^{-9}$ s)

So, $N \gg 0.5$ defects/Pb^{2+} or $N' \gg 10^{21}$ defects/cm^3.

According to previous reports, the defects density of perovskite single-crystal is usually 10^9-10^{10}/cm^3.[3,4] The defects density of 10^{21}/cm^3 (0.5 defects/Pb^{2+}) could be unlikely to occur in our materials. Thus, the power density of 23.5 W/cm^2 should be high enough to exclude that the broadband emission originate from permanent defects.

Supplementary references

1. Reshchikov, M. A. & Korotkov, R. Y. Analysis of the temperature and excitation intensity dependencies of photoluminescence in undoped GaN films. *Physical Review B* **64**, 115205 (2001).
2. Dohner, E. R., Jaffe, A., Bradshaw, L. R. & Karunadasa, H. I. Intrinsic white-light emission from layered hybrid perovskites. *J. Am. Chem. Soc.* **136**, 13154-13157 (2014).
3. Dong, Q. *et al.* Electron-hole diffusion lengths > 175 μm in solution-grown CH3NH3PbI3 single crystals. *Science* **347**, 967-970 (2015).
4. Shi, D. *et al.* Low trap-state density and long carrier diffusion in organolead trihalide perovskite single crystals. *Science* **347**, 519-522 (2015).
